# Dysregulated hepcidin response to dietary iron in male mice with reduced *Gnpat* expression

**DOI:** 10.1042/BSR20201508

**Published:** 2020-08-20

**Authors:** Gautam Rishi, Eriza S. Secondes, Kiran Asplett, Daniel F. Wallace, Lesa Ostini, Johannes Berger, V. Nathan Subramaniam

**Affiliations:** 1 School of Biomedical Sciences, Faculty of Health, Queensland University of Technology (QUT), Brisbane, Queensland, Australia; 2 Cell and Molecular Biology Department, QIMR Berghofer Medical Research Institute, Brisbane, Queensland, Australia; 3 Department of Pathobiology of the Nervous System, Center for Brain Research, Medical University of Vienna, Vienna, Austria

**Keywords:** GNPAT, hepcidin, Iron metabolism, iron overload, peroxisomes

## Abstract

Exome sequencing has identified the *glyceronephosphate O-acyltransferase* (*GNPAT*) gene as a genetic modifier of iron overload in hereditary hemochromatosis (HH). Subjects with *HFE* (*Homeostatic Iron Regulator*) p.C282Y mutations and the *GNPAT* p.D519G variant had more iron loading compared with subjects without the *GNPAT* variant. In response to an oral iron challenge, women with *GNPAT* polymorphisms loaded more iron as compared with women without polymorphisms, reinforcing a role for GNPAT in iron homeostasis. The aim of the present study was to develop and characterize an animal model of disease to further our understanding of genetic modifiers, and in particular the role of *GNPAT* in iron homeostasis. We generated an *Hfe/Gnpat* mouse model reminiscent of the patients previously studied and studied these mice for up to 26 weeks. We also examined the effect of dietary iron loading on mice with reduced *Gnpat* expression. *Gnpat* heterozygosity *in Hfe* knockout mice does not play a role in systemic iron homeostasis; *Gnpat^+/−^* mice fed a high-iron diet, however, had lower hepatic hepcidin (*HAMP*) mRNA expression, whereas they have significantly higher serum iron levels and transferrin saturation compared with wildtype (WT) littermates on a similar diet. These results reinforce an independent role of *GNPAT* in systemic iron homeostasis, reproducing in an animal model, the observations in women with GNPAT polymorphisms subjected to an iron tolerance test.

## Introduction

Iron homeostasis is a tightly regulated process involving several genes and alternate pathways [1–4]. Many of these genes were identified using classical genetic approaches in subjects with dysregulated iron metabolism. These studies linked mutations in *HFE* (Homeostatic Iron Regulator) [5], transferrin receptor 2 (*TFR2*) [6], hemojuvelin (*HJV*) [7], hepcidin (*HAMP*) [8] and ferroportin (*FPN/SLC40A1*) [9,10] to different forms of the genetic iron overload disorder, hereditary hemochromatosis (HH). The most common among these mutations is *HFE* p.C282Y, a substitution of tyrosine for cysteine in the HFE protein at position 282 (p.Cys^282^Tyr; p.C282Y) [5]. Up to 1 in 200 people of Caucasian origin are homozygous for this mutation [11], however a variability in phenotypic and clinical presentation of HH and accumulation of iron loading has been reported [12]. These observations suggest that other genetic, epigenetic or environmental factors play a role in the development of disease [13].

Using exome sequencing, we previously identified a variant (refSNP rs11558492) in the *glyceronephosphate O-acyltransferase* gene (*GNPAT* p.D519G) [14], which was associated with increased iron overload in patients homozygous for the *HFE* p.C282Y mutation. The *GNPAT* p.D519G variant has been identified as a cause of the peroxisomal disorder rhizomelic chondrodysplasia punctata (RCDP) 2 [15], where it was shown to lead to a 70% decrease in enzymatic activity of GNPAT [15]. Subsequently, a number of studies either supporting [16,17] or opposing [18–20] the role of *GNPAT* as a modifier of *HFE* hemochromatosis have been reported. Interestingly, it was also reported that in response to an oral iron tolerance test, subjects with *GNPAT* polymorphisms had significantly higher serum iron levels, transferrin saturation [21,22] and loaded more iron compared with subjects who did not have this polymorphism, even in the absence of the *HFE* mutations. In addition, the p.D519G variant of *GNPAT* was shown to be associated with lower serum hepcidin levels in response to increased transferrin saturation [22]. These studies have suggested an independent role for *GNPAT* in iron homeostasis where it may be involved directly or indirectly in regulating hepcidin in response to body iron levels.

*Gnpat^+/−^* mice fed a high fat, high cholesterol diet are more susceptible to lipid accumulation and were less responsive to fluvastatin treatment [23]. This suggests that plasmalogen biosynthesis may have a role to play in liver function. GNPAT is required in plasmalogen synthesis and is the first enzyme in this pathway [24]. Defects in GNPAT lead to disruption of membrane homeostasis due to the important role played by plasmalogens in membrane fluidity and lipid–lipid interactions [15]. Mutations in GNPAT lead to a type of rare genetic disorder, RCDP type 2. RCDP is a type of peroxisomal disorder which impairs the normal development of many parts of the body, leading to cranial abnormalities, hepatic dysfunction, visual and/or hearing loss, seizures and neural dysfunction [25] and there is evidence of disrupted iron homeostasis in patients suffering from defects in peroxisomes [26].

Based on our exome sequencing study [14] and recent mice studies [23,27] we examined the consequences of *Gnpat* heterozygosity in *Hfe* knockout mice at different ages and also in relation to dietary iron overload. While our study was being undertaken, a study reported that mice with total or hepatocyte-specific loss of *Gnpat* had normal systemic iron homeostasis [27] in the absence of *Hfe*, suggesting that *Gnpat* may not be a genetic modifier of iron homeostasis in mice.

## Methods

### Animal studies

Mice were housed under a 12:12-h light/dark cycle and were provided with food and water *ad libitum*. As previously observed by others, *Gnpat^−/*−*^* mice were not viable after birth; as this was also the case in our colonies, we used heterozygous *Gnpat* null mice for this study [23]. Heterozygous *Gnpat* null mice were initially generated by Prof Wilhelm Just, Universität Heidelberg, Heidelberg, Germany. [28]. Heterozygous *Gnpat* null (*Gnpat^+/−^*) male mice on a C57BL/6J x CD1.129 mixed background were bred to homozygous *Hfe* knockout (*Hfe^−/−^*) female mice on a C57BL/6J background, to obtain mice with the genotypes, *Hfe^+/−^/Gnpat^+/−^* or *Hfe^+/−^/Gnpat^+/+^*. Male and female littermates with the genotype, *Hfe^+/−^/Gnpat^+/−^*, were bred to obtain both the *Hfe* homozygous/*Gnpat* heterozygous (*Hfe^−/−^/Gnpat^+/−^*) and *Hfe* homozygous (*Hfe^−/−^*) male mice used for the present study. Mice were analyzed at 4, 10 and 26 weeks of age and fed normal chow (23200-12152, Specialty Feeds, Glen Forest, Western Australia).

Heterozygous littermates (*Hfe^+/−^/Gnpat^+/−^*) were also bred to generate *Gnpat* heterozygous (*Gnpat^+/−^*) and wildtype (WT) male mice, which were also used for a second study. Mice (4-week-old) were fed either a control (iron content: 68 mg/kg) (AIN93G, Specialty Feeds, Glen Forest, Western Australia) or a high-iron diet (0.25% carbonyl iron) for 1 week. The 0.25% carbonyl iron diet was prepared by mixing 2% carbonyl iron diet (SF07-082, Speciality Feeds) with iron-deficient diet (SF01-017, Speciality Feeds) in a 1:8 ratio. After 1 week, the mice were fasted for 6 h on the day of sacrifice and were anesthetized with ketamine (200 mg/kg, Provet Qld Pty Ltd, Northgate, QLD, Australia) and xylazine (10 mg/kg, Troy Laboratory Ltd, Smithfield, NSW, Australia). The anesthetized animals were then euthanized, killed by exsanguination and their tissues harvested for further analysis. Animals were housed and experiments performed at the QIMR Berghofer Medical Research Institute and the UQ Biological Research Facility at the Translational Research Institute, Brisbane, Australia. All subsequent tissue and molecular analysis took place at the Institute of Health and Biomedical Innovation (IHBI) at the Queensland University of Technology (QUT), Brisbane, Australia.

All animal studies were approved by the QIMR Berghofer Medical Research Institute (approval number: QIMR P1293), University of Queensland and Queensland University of Technology Animal Ethics Committees (approval number: QUT/TRI/511/16). Animals received ethical, humane and responsible care according to the criteria outlined in the ‘*Australian Code for the Care and Use of Animals for Scientific Purposes*, 2013’.

### Real-time PCR

RNA was isolated from mouse livers and spleens using TRIsure reagent (Bioline, Sydney, NSW, Australia). cDNA was prepared from 1 µg of total RNA using the SensiFAST cDNA synthesis kit (Bioline). For all investigated genes, real-time quantitative PCR (qPCR) was performed using the SensiFAST SYBR No-Rox kit (Bioline) under the following conditions: 5 min denaturation at 95°C, followed by 45 cycles of 95°C for 15 s, 60°C for 10 s and 72°C for 15 s. The relative expression of all target genes was calculated using the geometric mean of three reference genes: actin (*Actb*), hypoxanthine-guanine phosphoribosyl transferase (*Hprt*) and DNA directed RNA polymerase II subunit RPB1 (*Polr2a*). Primer sequences are presented in Table 1.

**Table 1:** Sequences of the primers used

Primer	Sequence (5′–3′)
*Actb-F*	GACGGCCAAGTCATCACTATTG
*Actb-R*	CCACAGGATTCCATACCCAAGA
*Hprt-F*	GGACTGATTATGGACAGGA
*Hprt-R*	GAGGGCCACAATGTGATG
*Polr2a-F*	AGCTGGTCCTTCGAATCCGC
*Polr2a-R*	CTGATCTGCTCGATACCCTGC
*Hamp-F*	AGAGCTGCAGCCTTTGCAC
*Hamp-R*	ACACTGGGAATTGTTACAGCATTTA
*Bmp6-F*	ATGGCAGGACTGGATCATTG
*Bmp6-R*	CCATCACAGTAGTTGGCAGCG
*Hfe-F*	CTGAAAGGGTGGGACTACATGTTC
*Hfe-R*	GGACACCACTCCCAACTTCGT
*Id1-F*	TTGGTCTGTCGGAGCAAAGCGT
*Id1-R*	CGTGAGTAGCAGCCGTTCATGT
*Smad7-F*	ACGGGAAGATCAACCCCGAG
*Smad7-R*	TTCCGCGGAGGAAGGTACAG
*Gnpat-F*	TCT CAG TGT TAC GAT GCG CT
*Gnpat-R*	GCC ACA AAG CCT CTG AGT TC

### Western blotting

Liver tissue (100 mg) was homogenized using Precelllys Evolution tissue homogenizer (Bertin Instruments, Montigny-le-Bretonneux, France) in a buffer with phosphatase inhibitors [29]. Protein lysate (25 µg) was electrophoresed on a Bolt™ 4–12% Bis-Tris Plus gels (Thermo Fisher Scientific, Massachusetts, United States). After transferring to a nitrocellulose membrane, the proteins were blocked with 10% non-fat milk in Tris buffer with 0.1% Tween-20 (TBST) for 2 h at room temperature, and incubated with primary antibodies: anti-Tfr2 [30] 1:10000, anti-pSmad (Cell Signaling Technology, Danvers, MA) 1:1000, anti-prohepcidin [31] 1:1000, anti-ferritin-H (Cell Signaling Technology) 1:1000, anti-actin (Sigma–Aldrich, St. Louis, Missouri) 1:20000 and anti-GAPDH (Merck Millipore, Kilsyth, Victoria, Australia) 1:200000 overnight at 4°C. After washing with TBST and incubated with anti-rabbit IgG-horseradish peroxidase (Invitrogen, Life Technologies) 1:10000, diluted in 10% non-fat milk in TBST for 1 h at room temperature. Excess secondary antibodies were washed off and the blots were incubated with chemiluminescent substrate (Lumina Forte; Merck Millipore) for 5 min. Blots were exposed to X-ray film (Fujifilm, Brookvale, NSW, Australia) and developed using the Minolta film processor (Konica Minolta Medical and Graphic, Tokyo, Japan).

### Histology

Tissues fixed with 10% formalin were processed, paraffin-embedded and sectioned by the Histology Laboratory, Central Analytical Research Facility, IHBI, QUT. Perls’ staining was performed as described [32]. The slides were then scanned using the Leica SCN400 slide scanner (Leica Microsystems, Wetzlar, Germany). The analysis was performed using the Digital Image Hub, image analysis software (Leica).

### Iron indices

Serum iron levels were determined using the commercial iron assay kit from Pointe Scientific (Canton, MI) [33]. Hepatic (HIC) and splenic (SIC) iron concentrations were measured in dried tissues using a colorimetric assay as previously described by Torrance and Bothwell [34].

### Statistical analysis

Statistical analysis was performed using GraphPad Prism 6 software (GraphPad Software, San Diego, CA). Statistically significant differences were determined using two-way analysis of variance (ANOVA). Tukey’s multiple comparison tests were performed to compare the differences between individual groups. *P*-values (<0.05) were considered to be statistically significant.

## Results

### Gnpat^+/−^ heterozygosity does not affect iron homeostasis in Hfe knockout mice

Exome sequencing studies have suggested that the *GNPAT* polymorphism (p.D519G) may be acting as a genetic modifier in patients with *HFE* hemochromatosis [14]. Male patients heterozygous for this polymorphism had more severe iron overloading as compared with *HFE* subjects without these polymorphisms [14]. In order to mimic these polymorphisms in mice we used the heterozygous *Gnpat*^+/−^ mouse model.

**Figure 1:**
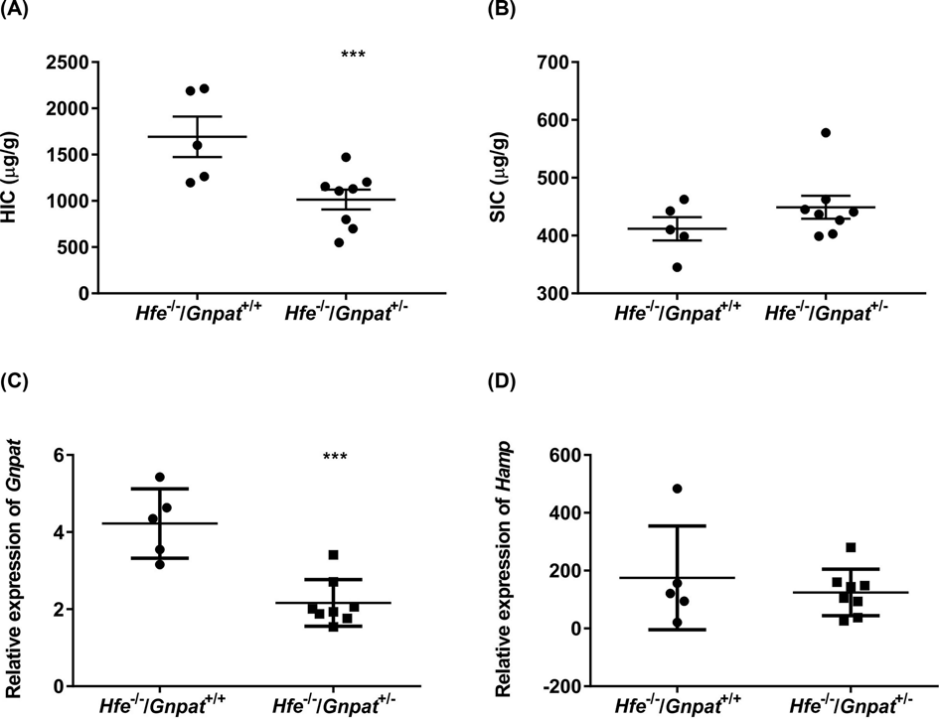
Iron parameters and expression of *Gnpat* and *Hfe* in 4-week-old *Hfe^−/−^* and *Hfe^−/−^/Gnpat^+/−^* mice The HIC (**A**), SIC (**B**), mRNA expression (relative to geometric mean of three reference genes: *Actb, Hprt* and *Polr2a*) of *Gnpat* (**C**) and *Hamp* (**D**) were measured in 4-week-old male *Hfe^−/−^* and *Hfe^−/−^/Gnpat^+/−^* male mice (*n*=5 and 8, respectively). Data are shown as dot plots, showing the mean and the standard error of the mean (SEM). Statistically significant differences (*t* test; *P*<0.001) are denoted as ***.

**Figure 2:**
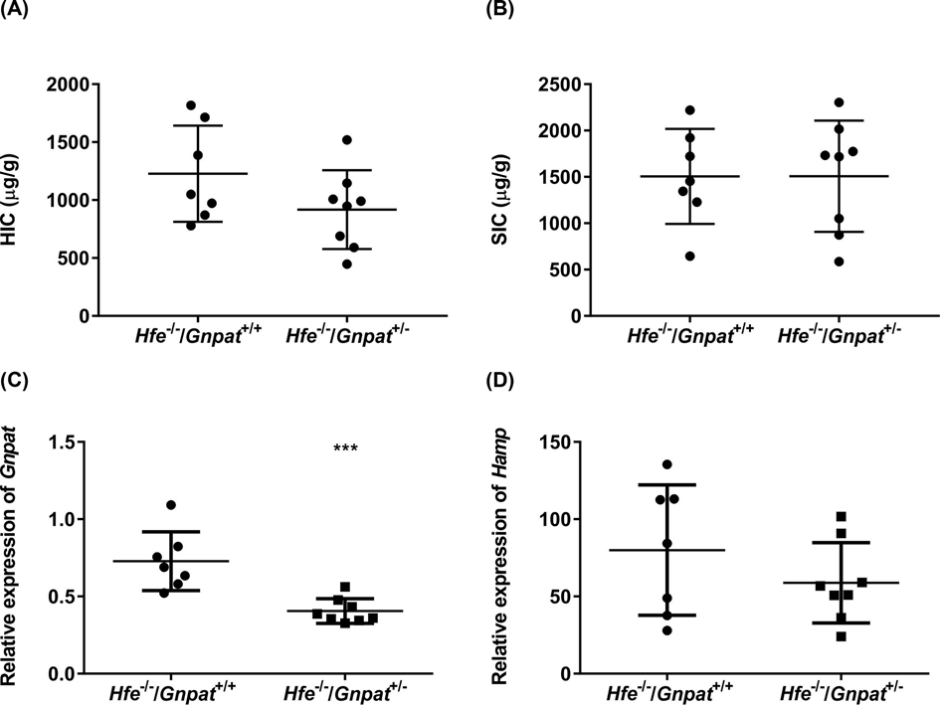
Iron parameters and expression of *Gnpat* and *Hfe* in 10-week-old *Hfe^−/−^* and *Hfe^−/−^/Gnpat^+/−^* mice The HIC (**A**), SIC (**B**) mRNA expression (relative to geometric mean of three reference genes*: Actb, Hprt and Polr2a*) of *Gnpat* (**C**) and *Hamp* (**D**) was measured in 10-week-old male *Hfe^−/−^* and *Hfe^−/−^/Gnpat^+/^*^−^ male mice (*n*=7 and 8, respectively). Data are shown as dot plots, showing the mean and the standard error of the mean (SEM). Statistically significant differences (*t* test; *P*<0.001) are denoted as ***.

**Figure 3:**
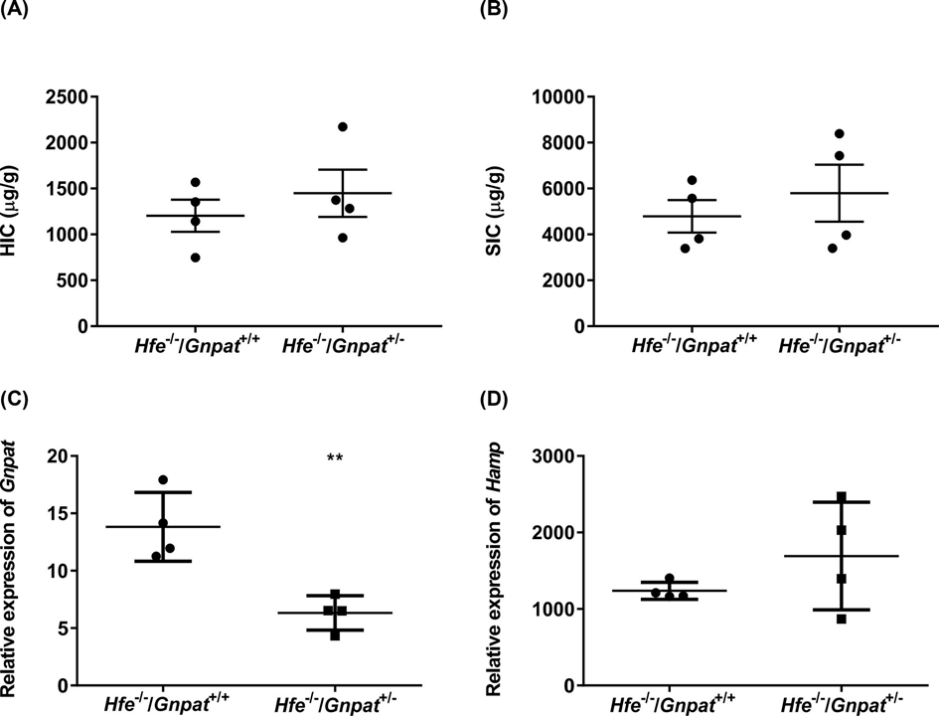
Iron parameters and expression of *Gnpat* and *Hfe* in 26-week-old *Hfe^−/−^* and *Hfe^−/−^/Gnpat^+/−^* mice The HIC (**A**), SIC (**B**), mRNA expression (relative to geometric mean of three reference genes: *Actb, Hprt and Polr2a*) of *Gnpat* (**C**) and *Hamp* (**D**) was measured in 26-week-old male *Hfe^−/−^* and *Hfe^−/−^/Gnpat^+/−^* male mice (both *n*=4). Data are shown as dot plots, showing the mean and the standard error of the mean (SEM). Statistically significant differences (*t* test; *P*<0.01) are denoted as **.

We examined the effect of *Gnpat* heterozygosity in *Hfe^−/−^* male mice by generating *Hfe^−/−^/Gnpat^+/−^* mice and comparing them with *Hfe^−/−^* littermates. These mice were all on the same background thus reducing the contribution of background to basal iron levels. Since our exome-sequencing study involved male patients and to avoid any gender-related differences, we used male mice for all our studies. Mice were analyzed at 4 (Figure 1), 10 (Figure 2) and 26 (Figure 3) weeks of age to examine any age-dependent effect of the loss of *Gnpat*. It is also known that iron accumulates with age in humans and mice [35]. We measured the HIC, SIC and relative mRNA expression of *Gnpat* and *Hamp* in the livers of these mice. As expected, the relative mRNA levels of *Gnpat* were significantly lower in the livers of the 4-, 10-, and 26-week-old male mice with *Gnpat* heterozygosity (*Hfe^−/−^/Gnpat^+/−^*) as compared with *Hfe^−/−^* male mice (Figures 1C–3C). The 4-week-old *Hfe^−/−^/Gnpat^+/−^* mice had significantly lower HIC as compared with the *Hfe^−/−^* mice (Figure 1A) but there were no differences in the splenic iron levels or *Hamp* mRNA levels in the livers of these mice (Figure 1B,D). There were no differences in HIC, SIC or relative mRNA levels of *Hamp* in the livers of the 10- and 26-week-old male mice (Figures 2 and 3). These results suggest that reduced Gnpat expression may not affect *Hfe* knockout mice in contrast with what might have expected from the patient studies, where the *GNPAT* p.D519G polymorphism was associated with more severe iron overload in *HFE* hemochromatosis patients [17].

### Effect of dietary iron challenge on Gnpat^+/−^ male mice

**Figure 4:**
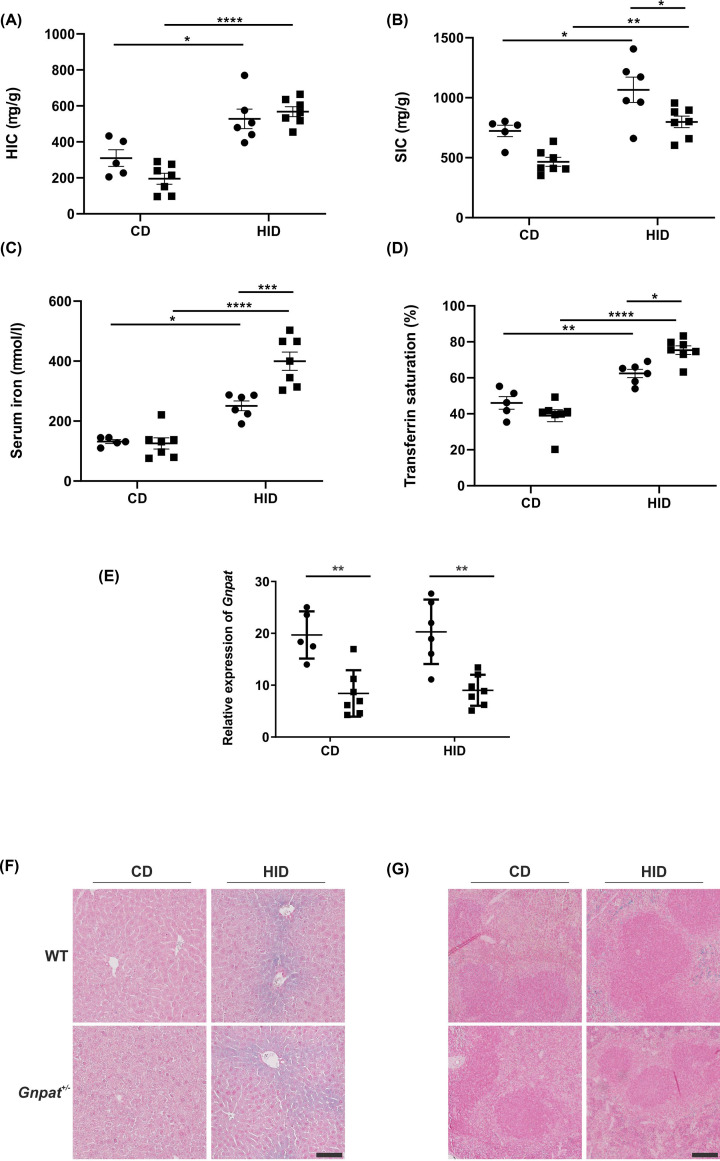
A comparison of iron indices in *Gnpat^+/−^* and WT littermates The HIC (**A**), SIC (**B**), total serum iron (**C**) and transferrin saturation (**D**) and mRNA expression (relative to geometric mean of three reference genes: *Actb, Hprt* and *Polr2a*) of *Gnpat* in the livers (**E**), were measured in 5-week-old (*n*=5–7) WT (●) and *Gnpat^+/−^* (▪) mice fed a control diet (CD) or 0.25% high-iron (HID) diet for 1 week. Data are shown as dot plots, showing the mean and the standard error of the mean (SEM). Statistically significant differences (two-way ANOVA using Tukey’s multiple comparison test) are denoted as *(*P*<0.05), **(*P*<0.01), ***(*P*<0.001) and ****(*P*<0.0001). Perls’ staining was performed on liver (**F**) and spleen (**G**) sections of 5-week-old (*n*=5–7) *Gnpat^+/−^* (Het) and WT mice fed a control diet (CD) or 0.25% high-iron (HID) diet for 1 week. This figure represents mice with the mean HICs in each group (scale bar = 100 µM).

While we were generating *Gnpat^+/−^* mice, two studies were published where subjects with GNPAT p.D519G were challenged with oral iron doses (iron tolerance test) [21,22]. To examine this effect in our animal models, 4-week-old male *Gnpat^+/−^* mice and WT littermates were fed a diet containing 0.25% carbonyl iron for one week. We chose this concentration of iron diet as we have shown that a 0.25% carbonyl iron containing diet is sufficient to induce maximal hepcidin response [36]. The *Gnpat^+/−^* male mice had similar HIC levels as compared with their WT littermates (Figure 4A). Mice fed a high-iron diet showed a significant increase in HIC, SIC, total serum iron and transferrin saturation as compared with mice fed a control diet with the same genotype. Interestingly, *Gnpat^+/−^* male mice fed a high-iron diet had significantly lower SIC as compared with WT mice on a similar diet (Figure 4B). The *Gnpat^+/−^* male mice fed a high iron diet also had significantly higher serum iron and transferrin saturation (Figure 4C,D) as compared with the control mice on a similar diet. These results indicate that there may be reduced iron deposition in the spleens of heterozygous *Gnpat^+/−^* male mice compared with WT littermates. Perls’ staining of the liver and spleen sections from the WT and *Gnpat^+/−^* mice fed either control or high-iron diet did not show any differences in the localization of iron (Figure 4F,G). We also examined the expression of *Gnpat* in the livers of these mice and as shown in Figure 4E, *Gnpat* mRNA expression does not change with increased iron.

**Figure 5:**
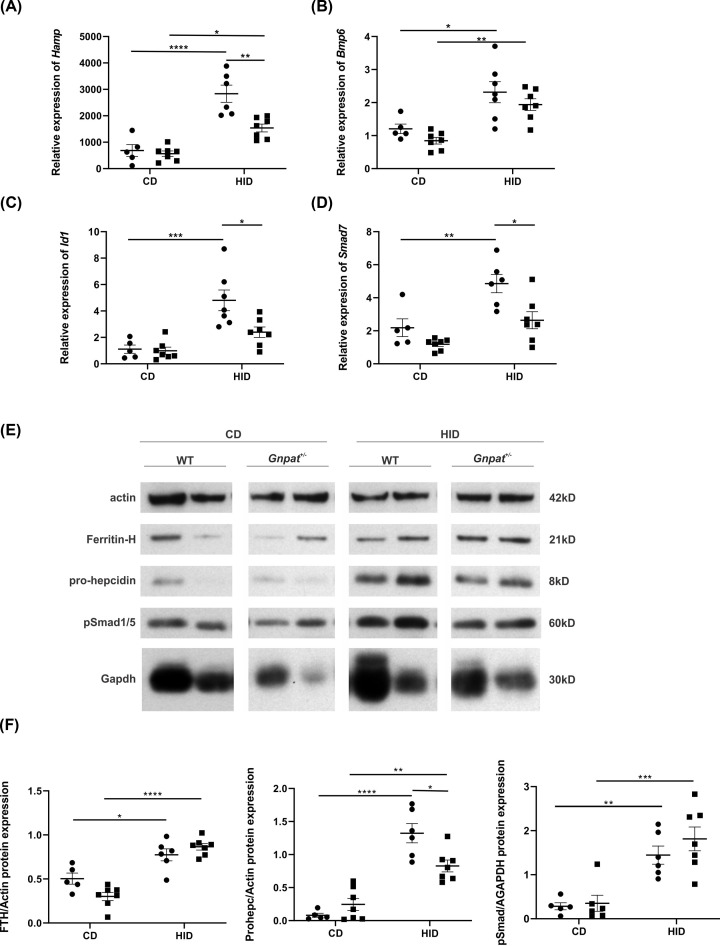
Signaling response in the livers of *Gnpat^+/−^* mice fed a 0.25% high-iron diet Relative mRNA expression (relative to geometric mean of three reference genes: *β-actin, Hprt* and *Polr2a*) of *Hamp* (**A**), *Bmp6* (**B**), *Id1* (**C**) and *Smad7* (**D**) was measured in the livers of 5-week-old (*n*=5–7) male WT (●) and *Gnpat^+/−^* (▪) fed a control diet (CD) or 0.25% high-iron diet (HID) for 1 week. Data are shown as dot plots, showing the mean and the standard error of the mean (SEM). Statistically significant differences (two-way ANOVA using Tukey’s multiple comparison test) are denoted as *(*P*<0.05), **(*P*<0.01), ***(*P*<0.001) and ****(*P*<0.0001). (**E**) Immunoblotting was performed using 25 µg homogenates from the livers of 5-week-old (*n*=5–7) male WT (●) and *Gnpat^+/−^* (▪) littermates fed a control diet (CD) or 0.25% high-iron diet (HID) for 1 week. Blots were probed with antibodies against pSMAD, H-ferritin, pro-hepcidin and β-actin and Gapdh, which were used as a loading control. The figure shows representative images of blots performed at least three times and on all mice from each group. (**F**) Quantification of the blots show that when mice were fed an HID, the pSmad, H-ferritin and pro-hepcidin expression increased and there was a significant difference in the levels of pro-hepcidin in the livers of WT (●) and *Gnpat^+/−^* (▪) mice fed a HID. Statistically significant differences (two-way ANOVA using Tukey’s multiple comparison test) are denoted as *(*P*<0.05), **(*P*<0.01), ***(*P*<0.001) and ****(*P*<0.0001).

The Bmp-Smad (sma and mothers and against decapentaplegic) pathway is known to be one of the most important signaling pathways involved in maintaining iron homeostasis. An increase in iron levels leads to an increase in bone morphogenetic protein 6 (Bmp6) which binds to its receptors and leads to activation of the Bmp-Smad pathway, resulting in increased expression of hepcidin [1,2]. Figure 5 shows that in response to an increase in dietary iron concentration, the levels of *Hamp, Bmp6*, inhibitor of DNA binding 1 (*Id1*) and SMAD family member 7 (*Smad7*) mRNA levels are increased in the livers of WT and *Gnpat^+/−^* mice, as expected. In response to increased iron levels, we saw a similar increase in *Bmp6* levels in the livers of both the WT and *Gnpat^+/−^* mice (Figure 5B). Interestingly, the relative expression of *Hamp, Id1* and *Smad7* (Figure 5A,C,D) were significantly lower in the *Gnpat^+/−^* mice fed a high-iron diet as compared with their WT littermates. Both *Id1* and *Smad7* are downstream molecules in the Bmp-Smad pathway and a reduction in their expression levels suggests that reduction in GNPAT may interfere with signaling in this pathway. These results suggest a defect in the Bmp-Smad signaling pathway in the *Gnpat^+/−^* mice. The protein levels of phospho-Smad (pSmad) 1/5 (Figure 5E,F) increased as the levels of iron increased in the diet. The pSmad levels in the livers of *Gnpat^+/−^* mice were similar to those of WT mice fed a high-iron diet. Similar to the mRNA expression results, the pro-hepcidin levels were significantly lower in the livers of *Gnpat^+/−^* mice fed a high-iron diet as compared with WT mice fed the same diet. An increase in the levels of iron in the diet, lead to an increase in ferritin levels as expected and the *Gnpat^+/−^* mice on a control diet had slightly lower ferritin, reflecting the lower HIC in these mice (Figure 4A).

## Discussion

*GNPAT* has been suggested to act as a genetic modifier of HH in patients with mutations in *HFE* [14,16,17]. Other reports using different cohorts failed to identify this similar correlation between *HFE* and *GNPAT* in patients with HH [18–20]. These varying results may be attributed to different study groups being used in the studies as well as other compounding factors, including but not limited to, alcohol consumption and the inclusion/exclusion criteria used to define the cohorts, which may lead to a low statistical power [37]. The original study used extreme phenotypes to differentiate between the two groups (patients with >1000 µg/ml of serum ferritin were considered as severe iron overload group and the mildly elevated group had serum ferritin values <300 µg/ml) [14]. Although this study had fewer numbers as compared with others [19,20,38], the other studies did not use extreme phenotypes as their case–control groups. This may also be one of the factors for differing results obtained in different cohorts as demonstrated by Besson-Fournier et al. [16], where they did not find any significant differences initially, but when they applied the same criteria as McLaren et al. [14] and examined the most severe males with serum ferritin levels >1000 µg/ml, the frequency of the rs11558492, allele G, was significantly enriched in this subset. Although controversial, the association of the rs11558492 allele G (p.D519G) with severe iron overload in *HFE* hemochromatosis patients cannot be completely ruled out.

In addition to the studies which analyzed the relationship between *GNPAT* and *HFE* in HH patients, a recent study also examined the relationship between iron and *GNPAT* polymorphisms in Taiwanese women [21]. Women with *GNPAT* polymorphisms had significantly higher fasting serum iron levels and after oral iron administration, both serum iron and transferrin saturation had increased significantly compared with women without *GNPAT* polymorphisms [21].

We fed 4-week-old male *Gnpat^+/−^* mice and their WT littermates a high-iron diet containing 0.25% carbonyl iron for 1 week. The *Gnpat^+/−^* male mice fed a high-iron diet had a significantly decreased SIC as compared with the WT littermates, suggesting that there may be a dysregulation in iron homeostasis in *Gnpat^+/−^* male mice. It has been shown that iron homeostasis in mice can be affected by strain-specific differences [39,40]. However, we minimized any effects of this by using WT littermate controls for these studies.

In our study, we did not observe any differences between the Gnpat^+/−^ and WT mice in terms of Bmp6 levels, as also noted by An et al. [27]. Interestingly, mRNA expression of *Id1* and *Smad7*, molecules downstream in the pathway, were lower in the livers of *Gnpat^+/−^* mice fed a high-iron diet as compared with the WT littermates. The fact that there are no differences in the levels of upstream molecules (*Bmp6*) suggests that *Gnpat* may not be involved in sensing the levels of iron, but may be involved in the effective relaying of signaling either directly or indirectly, as suggested by the lower levels of mRNA expression of other downstream molecules involved in the Bmp-Smad pathway. An et al. [27] also observed a reduced hepcidin response to BMP6 treatments in primary murine hepatocytes where *Gnpat* had been targeted using siRNA.

*Gnpat^+/−^* mice fed an iron-rich diet have significantly increased serum iron and transferrin saturation as compared with their control littermates, this is similar to what was observed in the female subjects in the Taiwanese study, where subjects with *GNPAT* polymorphisms had significantly higher serum iron and transferrin saturation when administered oral iron [21]. These results suggest that Gnpat may be involved in some way in regulation of iron transport. Indeed, previous studies have shown that the rate of endocytosis and the rate of labeled transferrin uptake was reduced in skin fibroblasts from patients with GNPAT mutations as compared with control cells [15], which suggests a reduced or slower uptake of iron. It was also shown that the rate of transferrin recycling was also slower in the fibroblasts from patients with *GNPAT* mutations [15]. These results are different from the observations made by An et al. [27] where dietary iron loading of *Gnpat* KO or *Hfe/Gnpat* double knock mice did not show any differences in systemic iron homeostasis suggesting *Gnpat* does not act as a genetic modifier in *Hfe* KO mice. The differences between the two studies could be due to the concentration of dietary iron used; An et al. used approximately 0.8% carbonyl carbon in their diet whereas in our study we used 0.25% iron. We have previously shown that 0.25% iron is sufficient to elicit a hepcidin response in the livers of WT mice and increased iron concentrations only lead to an accumulation of iron in the liver [36]. In this study we examined male mice, whereas An et al. examined the effect of total loss of Gnpat in female mice. Previous studies have shown that gender can have an effect on plasmalogen functions [41] and the effects of gender on systemic iron homeostasis are well known [42–44]. Taken together, these differences in the models may be able to explain the difference in the observations made in our study and An et al. [27].

Some of our data, specifically with the mice with *Gnpat* heterozygosity and *Hfe* suggests that *Gnpat* may not play a role as a genetic modifier in *HFE* hemochromatosis as also suggested by An et al. [27]. However, in our study the HIC was significantly lower in the *Hfe* KO mice with *Gnpat* heterozygosity at 4 weeks of age as compared with *Hfe* KO mice, which suggests that there may be some dysregulation in iron uptake in these mice. In our recent study we showed that defects in peroxisomal function can lead to defects in the expression of multiple membrane proteins involved in iron homeostasis [45], including transferrin receptor 1 (TFR1) the receptor involved in the uptake of transferrin bound iron, since Gnpat is an essential enzyme required for peroxisomal function, these observations combined with previous results showing defects in iron recycling in fibroblasts with GNPAT defects [15] may explain the lower iron levels in the livers of mice with *Gnpat* heterozygosity. The reduced response to BMP6 in primary hepatocytes lacking Gnpat, as observed by An et al. [27], and the observations from the dietary iron model in our study, suggest that Gnpat may play a role in iron homeostasis.

The significantly lower SIC, higher the transferrin saturation and serum iron levels in the *Gnpat^+/−^* mice is also similar to the hemochromatosis phenotype observed in *Hfe* and *Tfr2* KO mice, where the KO mice have lower SIC as compared with their WT littermates suggesting increased release of iron from the macrophages as a result of reduced hepcidin levels.

The results from our study and An et al. [27] suggest that Gnpat may not be a genetic modifier in HFE-hemochromatosis, however, this study examined the effect of heterozygosity of *Gnpat*, and not the effect of the *GNPAT p.D519G* variant believed to be associated with iron overload in HFE HH patients, and the possibility of this variant having an effect on HFE function cannot be completely ruled out based on the present study.

In a recent study, we have also shown that defects in peroxisomes, the organelles where GNPAT is functional, affect iron homeostasis [45]. The biochemical defects due to the dysfunctional peroxisomes lead to several changes in the liver which include affecting the expression of membrane proteins involved in maintain iron homeostasis. These results taken together with the observations made in this study suggest a role for genes involved in plasmalogen synthesis in liver function including iron homeostasis.

## Open Access

Open access for this article was enabled by the participation of Queensland University of Technology in an all-inclusive *Read & Publish* pilot with Portland Press and the Biochemical Society under a transformative agreement with CAUL.

## Abbreviations

Bmp6: bone morphogenetic protein 6
GNPAT: glyceronephosphate O-acyltransferase
HAMP: hepcidin
HFE: homeostatic iron regulator
HH: hereditary hemochromatosis
HIC: hepatic iron concentration
Id1: inhibitor of DNA binding 1
pSmad: phospho-Smad
RCDP: rhizomelic chondrodysplasia punctata
SIC: splenic iron concentration
Smad: sma and mothers and against decapentaplegic
Smad7: SMAD family member 7
TBST: Tris buffer with 0.1% Tween-20
TFR2: transferrin receptor 2
WT: wildtype
